# Mechanical Properties and Carbon Emission Characteristics of Loess Stabilized with Multi-Source Solid Waste Cementitious Materials

**DOI:** 10.3390/ma19153191

**Published:** 2026-07-26

**Authors:** Bentian Yu, Yuting Cai, Leyu Niu, Hao Wang, Dongze Xia, Xinzhu Li

**Affiliations:** 1 School of Civil Engineering, Lanzhou Jiaotong University, Lanzhou 730070, China; 13919012035@163.com (Y.C.); 15847469765@163.com (H.W.); 18345835507@163.com (D.X.); 18995682827@163.com (X.L.); 2 School of Engineering, Cardiff University, Cardiff CF24 3AA, UK; niuleyu2007@126.com; 3 Lanzhou Center for Disease Control and Prevention, Lanzhou 730030, China

**Keywords:** carbon emissions, multi-source solid waste, alkali activation, stabilized loess, mechanical properties, improvement mechanism

## Abstract

**Highlights:**

- The optimal mixing formula for alkali-activated stabilized loess contains 15% multi-source solid waste, 3% NaOH, and 3.2% sodium silicate by mass of solid waste.
- Physical filling and alkali-triggered gelation synergistically improve soil compaction, mechanical performance, and water stability.
- Solid waste-stabilized loess cuts carbon emissions by 74.1~88.9% versus cement-stabilized loess.
- Combined physical filling and alkali-activated cementation is an effective technical route to upgrade loess mechanical and waterproof properties.
- Alkali modulus needs reasonable control to balance early pore refinement and long-term pore stability of stabilized loess.
- Using multi-source solid waste replaces ordinary cement for loess treatment, drastically lowering construction’s carbon footprint and realizing solid waste resource recycling.

**Abstract:**

To address the high carbon emissions generated during the production of cement, lime, and other traditional soil stabilizers and to promote the resource utilization of industrial solid waste, this study proposed a low-carbon loess stabilization scheme using tuff powder (TP), fly ash (FA), and ground granulated blast-furnace slag (GGBS) activated by alkaline solutions to fully substitute conventional cement and lime. A series of macroscopic tests, including unconfined compressive strength, water immersion, and triaxial shear tests, were carried out on stabilized loess. Combined with microcharacterization including X-ray diffraction (XRD), scanning electron microscopy (SEM), and nuclear magnetic resonance (NMR) spectroscopic testing, this study systematically evaluated the mechanical performance, water stability, and microstructural evolution of stabilized loess and quantified its global warming potential (GWP). Macroscopic test results reveal that the composite binder consisting of 15% multi-source solid waste (TP:FA:GGBS) = (1:1:3), 3% NaOH, and 3.2% Na_2_SiO_3_ (relative to solid waste mass) delivers the optimal comprehensive performance of stabilized loess. Alkali activation significantly accelerates early strength development, and both compressive and shear strengths are markedly improved compared with samples treated solely with solid waste. Furthermore, decreasing the activator modulus further enhances mechanical properties and water resistance. Microscopic characterizations demonstrate that alkali activation stimulates the pozzolanic reaction of active components in solid waste, generating cementitious gels that bind soil particles and unreacted solid waste to form dense network matrices. As a fine filler, TP also acts as a nucleation sites for hydration product crystallization, which facilitates the formation of cementitious phases.

## 1. Introduction

Loess is characterized by loose granular structures, weak interparticle cohesion, and prominent collapsibility, leading to poor water resistance and freeze-thaw durability [1,2]. When utilized as subgrade fill, loess easily induces engineering hazards such as differential settlement, slope failure, frost heave, and water-induced collapse [3], which severely threatens infrastructure safety. Therefore, loess must be stabilized prior to engineering application. At present, cement and lime are the most widely adopted stabilizers for loess improvement in practical projects [4]. Nevertheless, the manufacturing of cement and lime releases massive CO_2_ and exacerbates the greenhouse effect [5]. Meanwhile, China maintains a high annual output of industrial solid waste accompanied by a low comprehensive utilization rate; massive stockpiling of such waste poses severe environmental risks.

In recent decades, numerous researchers have explored the feasibility of using industrial solid waste for loess stabilization, mainly focusing on partial replacement of cement/lime with solid waste. Zhang et al. [6] reported that mixtures of fly ash and lime exhibit superior structural strength compared with single lime-stabilized soil within a 30–180 d curing period. Zhang [7] further verified that cement-fly ash blends significantly improve the freeze-thaw resistance of loess. However, partial substitution only reduces carbon emissions to a limited extent and can’t fundamentally resolve the high-carbon drawback of cement production.

Subsequent studies have investigated the full replacement of cement and lime with industrial solid waste. Mozejko et al. [8] compared the mechanical performance of steel slag-stabilized loess with cement/lime-stabilized loess and found that the unconfined compressive strength of stabilized loess with optimal steel slag dosage was lower than that of cement/lime-stabilized loess, which was attributed to the relatively low pozzolanic reactivity of steel slag. To enhance the reactivity of solid waste blends, Mohammadi et al. [9] explored the stabilization performance of various fly ash types activated by recycled alkaline solutions and concluded that high-calcium fly ash possesses higher activation potential than low-calcium fly ash. Turan et al. [10] pointed out that alkaline activators can boost fly ash reactivity, thus elevating both early and long-term strengths of stabilized soil. Jia [11] investigated the effects of water glass modulus and alkali content on FA-GGBS-stabilized loess and observed that compressive strength continuously rises with decreasing water glass modulus and increasing alkali content. When the water glass modulus was 1.5 and alkali dosage reached 1.5%, the 28 d compressive strength of stabilized loess reached 14.9 MPa, 33.7 times higher than raw loess. Hamed et al. [12] optimized the mix ratio of FA-GGBS geopolymer for expansive soil stabilization, obtaining an optimal formulation of 38% solid waste (24% FA + 14% GGBS) activated by 25.75% NaOH (12 mol/L). Although existing literature has proven the technical viability of all-solid-waste soil stabilization, the overall mechanical performance of such materials is generally inferior to traditional cement-based stabilizers, restricting their large-scale engineering promotion.

Current research on alkali-activated full-solid-waste loess stabilization predominantly relies on FA and GGBS [13]. Nevertheless, extensive consumption of FA and GGBS in concrete projects and uneven regional resource distribution limit their supply for loess improvement [14]. In addition, emerging types of industrial solid waste are continuously generated and accumulated, causing severe environmental pollution. Accordingly, scholars have begun to explore the synergistic effect of multiple solid waste fractions to partially replace conventional FA/GGBS. The FA-steel slag system proposed by Chai [15] and the marble powder-modified clay reported by Zeybek et al. [16] both confirm the synergistic enhancement potential of multi-source solid waste blends. TP is a fine by-product collected by electrostatic precipitators during manufactured sand production. Rich in silica and alumina, TP exhibits moderate pozzolanic reactivity and can serve as a potential supplementary cementitious material to substitute FA and GGBS [17]. Furthermore, fine TP particles fill intergranular voids of loess to form micro-aggregate structures and improve soil compaction. TP also acts as nucleation sites for hydration product crystallization to accelerate the formation of cementitious phases. The combined physical filling of TP and chemical cementation of alkali-activated gels produce a synergistic stabilization effect on loess. However, few studies have focused on TP-FA-GGBS composite systems for loess stabilization, and their synergistic mechanisms and macroscopic geotechnical properties remain insufficiently clarified.

Against this background, this paper proposes a ternary multi-source solid waste binder (TP-FA-GGBS) fully replacing cement and lime under dual activation of NaOH and Na_2_SiO_3_. Macroscopic mechanical tests and microstructural characterizations were conducted on alkali-activated stabilized loess. Through unconfined compressive strength, water immersion, and triaxial compression tests, the influences of solid waste dosage and activator modulus on mechanical properties and water stability were revealed. Combined with microcharacterization including XRD, SEM-EDS, and NMR spectroscopic testing, this paper clarifies the evolution laws of pore structure, mineral phase composition, and micromorphology and further reveals the macro-micro coupled stabilization mechanism of loess modified by multi-source solid wastes. The research outcomes provide theoretical support and technical references for the resource recycling of novel solid waste such as TP in loess subgrade treatment.

## 2. Materials and Methods

### 2.1. Raw Test Materials

**Figure 1:**
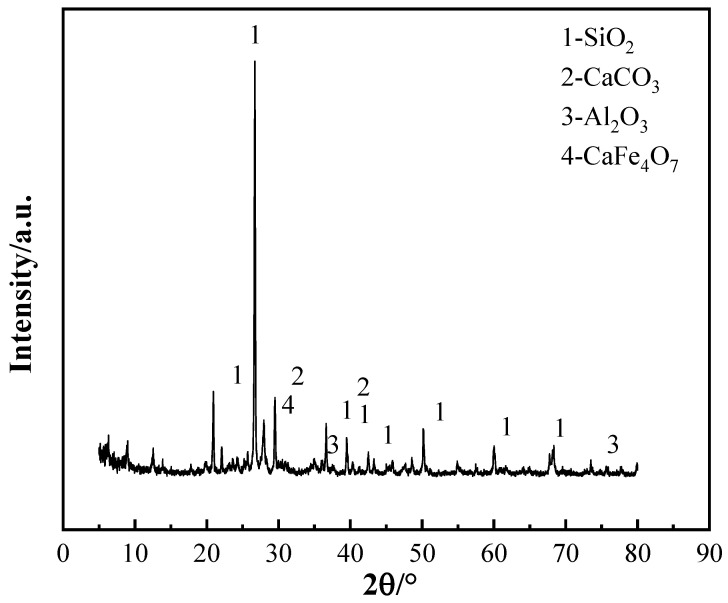
XRD mineralogical pattern of raw loess.

Raw loess was sampled from Anning District, Lanzhou, Gansu Province, China, presenting a yellowish-brown appearance. Basic physical indices were measured in accordance with the Standard for Geotechnical Testing Methods (GB/T 50123-2019 [18]), as summarized in Table 1. The XRD pattern of raw loess is illustrated in Figure 1; the primary mineral component is SiO_2_, with minor fractions of Al_2_O_3_ and CaCO_3_.

TP is an industrial by-product collected by electrostatic precipitators during the crushing process of manufactured sand produced from natural volcanic tuff. TP, FA, and GGBS used in this test were all provided by Gansu Construction Investment Group Co., Ltd., Lanzhou, China, and their primary chemical oxide components are summarized in Table 2.

**Figure 2:**
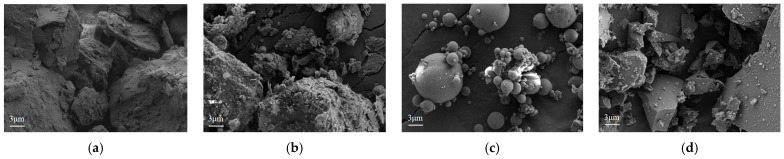
SEM micromorphology of raw materials: (**a**) Raw loess; (**b**) TP; (**c**) FA; (**d**) GGBS.

**Figure 3:**
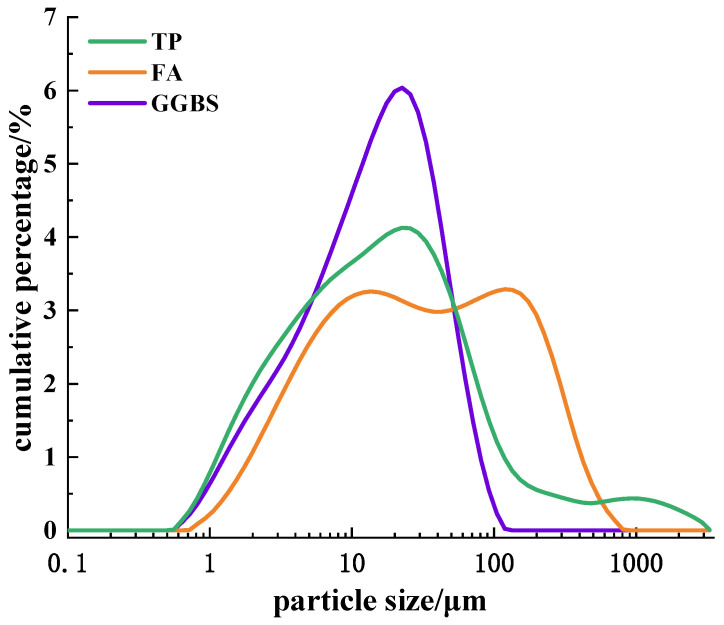
Particle size distribution curve of TP, FA, and GGBS.

Figure 2 displays the SEM micromorphology of all raw materials. Raw loess exhibits a multi-scale granular structure with rough particle surfaces. TP exists as a gray powder with angular and coarse particles. Class II F fly ash is dominated by smooth spherical microspheres. S95-grade GGBS presents an off-white appearance and consists of irregular smooth particles. Figure 3 illustrates the particle size distribution curves of the three solid wastes.

Solid NaOH and Na_2_SiO_3_·9H_2_O were adopted as alkaline activators in this study. Analytical-grade NaOH with purity exceeding 96% and carbonate impurity below 1.5% was supplied by Shanghai Guangnuo Chemical Technology Co., Ltd., Shanghai, China. The sodium silicate solution was purchased from Tianjin Baishi Chemical Co., Ltd., Tianjin, China, which contained 19.3–22.8% Na_2_O and exhibited an initial modulus of 1.03 ± 0.03.

### 2.2. Mix Proportion Design

Based on the research results of References [19,20], the response surface methodology (RSM) was first adopted to optimize and determine the basic mixing ratio of multi-source solid waste and alkaline activators, and then the formal mixing ratios were designed in this work. The ternary solid waste blend was compounded with TP:FA:GGBS = 1:1:3 by mass and incorporated into loess at dosages of 5%, 8%, 10%, 12%, 15%, and 20% relative to total dry loess mass. For activator preparation, NaOH and Na_2_SiO_3_ were dissolved in deionized water at fixed mass ratios of 2% and 3.2% of solid waste mass to prepare composite alkaline solutions. To investigate the effect of activator modulus (molar ratio n(SiO_2_)/n(Na_2_O)), the sodium silicate dosage was fixed, while NaOH content was adjusted to 1%, 2%, 3%, and 4% of solid waste mass to modulate modulus values. Detailed mix proportions are shown in Table 3.

### 2.3. Test Method

**Figure 4:**
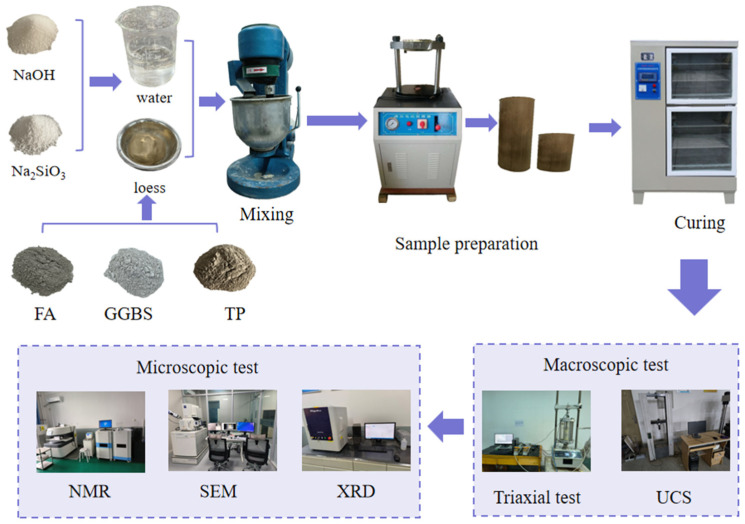
Schematic diagram of the full experimental workflow.

Raw loess was oven-dried, crushed, and sieved through a 2 mm sieve, then sealed for standby. Compaction tests were performed using a heavy Z1 compactor with a 4.5 kg hammer, a compaction work of 2659 kJ/m^3^, and 25 blows per specimen, in accordance with the Railway Soil Test Regulations (TB 10102-2023 [21]). Soil samples were prepared at moisture contents of 9%, 11%, 12%, 13%, and 15%, then sealed and cured for 24 h to achieve uniform water distribution. Pre-weighed solid waste blends were mixed homogenously with wetted loess, and compaction curves were plotted to obtain the optimum water content (OMC) and maximum dry density of each mixture; all calculations complied with the Railway Soil Test Regulations (TB 10102-2023). The total mixing water dosage (including liquid alkaline activator) was uniformly controlled at the OMC of the multi-source solid waste composite system, where water contained in the activator solution was subtracted from the total added water. For alkali-activated groups, loess was first blended with 80% of the target optimum water content and sealed for 24 h to ensure uniform water distribution. The remaining 20% water was used to dissolve NaOH and Na_2_SiO_3_ for alkaline activator preparation, which was subsequently mixed with solid waste and pre-wetted loess. Cylindrical specimens were fabricated via static compaction. Specimens for unconfined compressive strength (UCS) and water-immersed compressive strength measurements were 50 mm in diameter and 50 mm in height, with six parallel replicates prepared for each testing group. Triaxial test specimens were manufactured to dimensions of 50 mm diameter × 100 mm height, and six replicate samples were produced for each confining pressure condition. Specimens for NMR analysis were uniformly sized at 50 mm × 50 mm. Following compaction, all specimens were wrapped in plastic film and cured within a standard constant temperature and humidity chamber until the designated curing age was achieved. For XRD and SEM characterizations, fractured specimens obtained from unconfined compressive strength tests were oven-dried at 60 °C over a 24 h period. Flat fracture surfaces were selected for SEM morphological observation. The remaining broken fragments were pulverized and sieved using a 0.075 mm mesh sieve to prepare powders for XRD mineral phase identification. A complete flowchart illustrating the full experimental procedure is provided in Figure 4.

## 3. Experiment Results and Analysis

### 3.1. Compaction Characteristics

Compaction test results of raw loess and solid waste stabilized loess are summarized in Table 4. After incorporating multi-source solid waste, the maximum dry density of stabilized loess first rises and then declines with increasing solid waste dosage. This trend is mainly attributed to the fine solid waste particles filling intergranular voids of loess at moderate dosages, densifying the soil matrix and elevating dry density. When solid waste dosage exceeds the optimal filling threshold, excessive fine particles disrupt the compacted skeleton, leading to a reduction in maximum dry density. The variation of optimum moisture content follows a similar trend but is less sensitive to solid waste dosage. At a solid waste dosage of 15%, stabilized loess achieves the maximum dry density and optimum moisture content, which are 8.0% and 1.5% higher than raw loess, respectively.

### 3.2. Unconfined Compressive Strength

**Figure 5:**
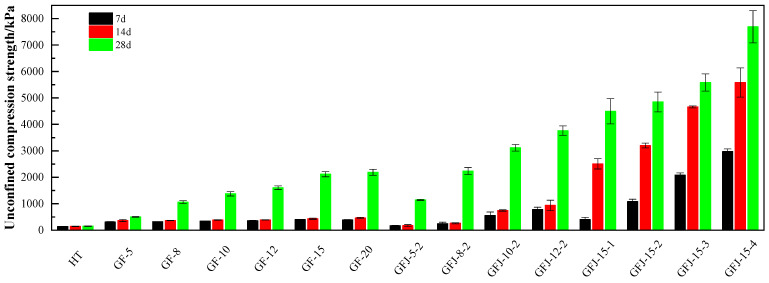
Evolution of unconfined compressive strength of stabilized loess with curing age.

Figure 5 illustrates the unconfined compressive strength evolution of raw loess and solid waste-stabilized loess at different curing ages. The compressive strength of solid waste stabilized loess increases monotonically with rising solid waste dosage. At a 15% solid waste dosage (GF-15), the 28 d compressive strength is three times that of raw loess (HT). Further increasing dosage to 20% (GF-20) yields negligible strength growth, indicating a strength plateau for non-activated solid waste stabilized loess. Non-activated solid waste-stabilized loess exhibits low strength before 14 d curing, with rapid strength gain observed between 14 d and 28 d. This delayed strength development originates from the low inherent pozzolanic reactivity of unactivated solid waste, which slows the hydration reaction rate. For alkali-activated groups, GFJ-5-2 and GFJ-8-2 show insignificant strength improvement due to insufficient solid waste to generate adequate hydration products. When solid waste dosage exceeds 10%, the 28 d compressive strength of alkali-activated samples is 1.3, 1.4, and 1.3 times higher than the corresponding non-activated GF-10, GF-12, and GF-15 groups, respectively. Alkali activation significantly accelerates early strength development: taking GFJ-15-2 as an example, its 7 d compressive strength is 6.9 times higher than raw loess and 1.7 times higher than GF-15 (non-activated). The 28 d compressive strength of GFJ-15-2 reaches approximately 30 times that of raw loess and 1.3 times that of GF-15. Comparing GFJ-15-1, GFJ-15-2, GFJ-15-3, and GFJ-15-4, compressive strength continuously improves with decreasing activator modulus. The 7 d compressive strength of GFJ-15-4 is six times that of GFJ-15-1, demonstrating that a high-alkaline environment facilitates early strength gain of alkali-activated multi-source solid waste-stabilized loess.

### 3.3. Water Immersion Test and Water Stability

**Figure 6:**
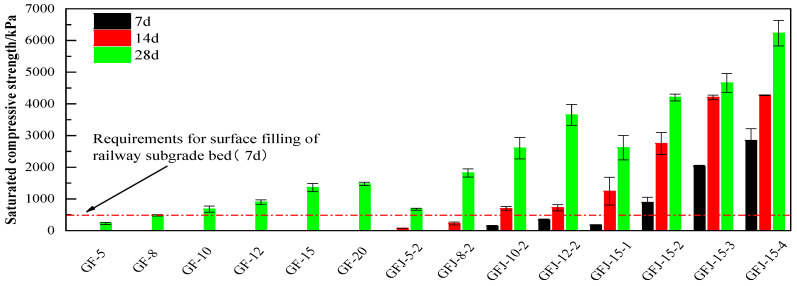
Variation of saturated unconfined compressive strength of stabilized loess.

**Figure 7:**
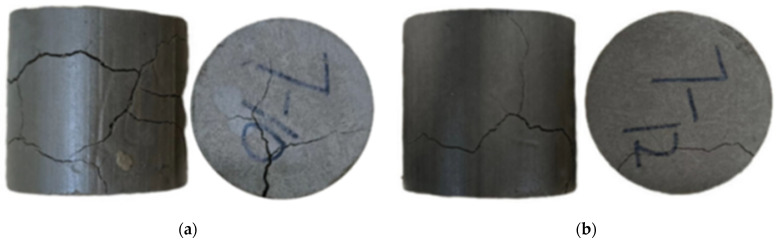
Crack morphology of stabilized loess after water immersion: (**a**) GFJ-10-2; (**b**) GFJ-12-2.

Water immersion test results are displayed in Figure 6. Non-activated solid waste-stabilized loess exhibits poor early water resistance; saturated compressive strength equals zero within the first 14 d curing, and measurable saturated strength only forms after 28 d. For alkali-activated systems, saturated compressive strength remains zero at 7 d when solid waste dosage is below 10%. At 10–12% solid waste dosage, 7 d saturated strength stays at a low level. When solid waste dosage increases to 15% (GFJ-15-2), the 7 d saturated unconfined compressive strength reaches 891 kPa, which meets the subgrade fill strength requirement specified in the Code for Design of Railway Earth Structure (TB 10001-2016 [22]). Saturated compressive strength rises continuously with decreasing activator modulus, accompanied by drastically improved early water stability. Increasing NaOH dosage from 1% to 4% boosts the 7 d saturated strength by 15 times. Low-dosage, short-curing alkali-activated specimens develop obvious cracks after immersion (Figure 6). This phenomenon arises from accelerated hydration of incompletely reacted solid waste binders under water saturation, inducing local volumetric expansion and stress concentration to propagate cracks [23]. With higher solid waste dosage and longer curing age, crack width gradually decreases and ultimately disappears (Figure 7).

### 3.4. Triaxial Shear Strength Characteristics

**Figure 8:**
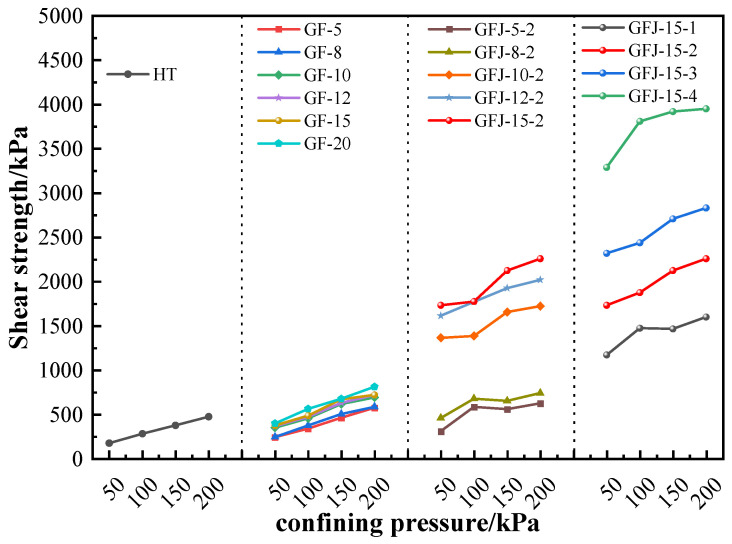
Shear strength of stabilized loess under different confining pressures.

Figure 8 presents the shear strength of raw loess and stabilized loess under varying confining pressures. The shear strength of all soil samples increases with elevated confining pressure. For non-activated solid waste-stabilized loess, shear strength slightly rises with solid waste dosage, indicating limited influence of solid waste content on shear performance. In contrast, alkali activation significantly enhances shear strength. At solid waste dosages below 10%, GFJ-5-2 and GFJ-8-2 show shear strength comparable to GF-5 and GF-8. When solid waste dosage exceeds 10%, the shear strength of GFJ-10-2 under 150 kPa confining pressure is 1.5 times that of GFJ-8-2 and 1.7 times that of non-activated GF-10, proving that alkali-activated solid waste dosage must exceed 10% to achieve prominent early shear strength improvement at 7 d curing. Further analysis reveals that shear strength increases with decreasing activator modulus; under 150 kPa confining pressure, GFJ-15-4 delivers 1.7 times the shear strength of GFJ-15-1.

**Figure 9:**
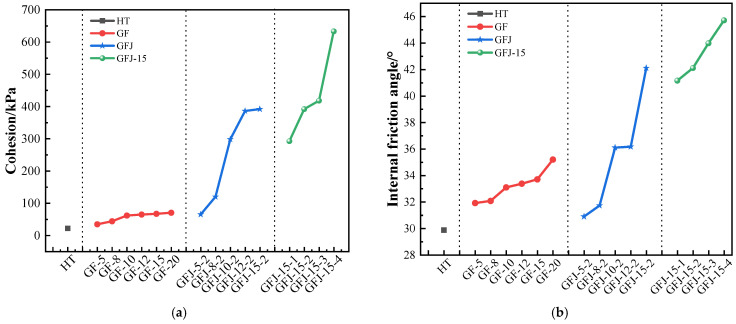
Shear strength indices of stabilized loess: (**a**) Cohesion; (**b**) Internal friction angle.

Figure 9 illustrates the evolution of cohesion and internal friction angle for each sample group. Non-activated solid waste stabilized loess shows negligible cohesion increment relative to raw loess, while the internal friction angle rises moderately. Increasing solid waste dosage barely improves cohesion but greatly elevates the internal friction angle. The addition of solid waste increases particle surface roughness and strengthens interparticle mechanical interlocking, which dominates friction angle growth. Meanwhile, limited hydration products are generated in non-activated systems, resulting in insignificant cohesion enhancement. Alkali activators accelerate solid waste hydration and generate abundant cementitious gels, which greatly strengthen interparticle bonding and interlocking. Both cohesion and internal friction angle of alkali-activated stabilized loess outperform raw loess and non-activated solid-waste-stabilized loess. Cohesion and friction angle increase with higher solid waste dosage, and both indices rise as activator modulus declines, with cohesion showing a more sensitive response. Reducing activator modulus substantially increases the yield of cementitious binders yet imposes minor effects on particle interlocking, leading to mild growth of the internal friction angle.

## 4. Stabilization Mechanism

### 4.1. NMR Pore Structure Analysis

**Figure 10:**
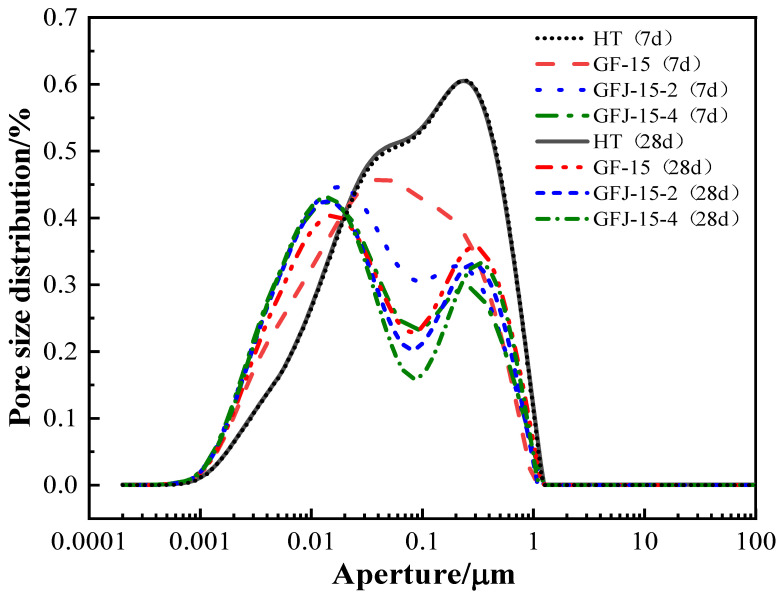
Pore size distribution curves of raw loess and stabilized loess from NMR tests.

Figure 10 shows NMR pore size distribution curves of raw loess and stabilized loess at different curing ages. The pore distribution curves of HT at 7 d and 28 d nearly overlap, indicating that raw loess maintains a stable high-porosity structure dominated by large pores throughout curing. After incorporating 15% non-activated solid waste (GF-15), the characteristic peak of the 7 d curve shifts leftward with reduced peak intensity, as solid waste particles fill loess voids and reduce overall porosity and pore diameter. Extending curing age to 28 d induces further left shift of the characteristic peak, demonstrating that continuous solid waste hydration products further refine pore structures. After alkali activation, the 7 d curve of GFJ-15-2 exhibits a more significant left shift and peak attenuation compared with GF-15, which verifies that alkali activation accelerates gel formation and realizes secondary pore filling [24]. At 28 d, the peak intensity further decreases while the peak position remains nearly unchanged, implying that later-stage curing mainly reduces pore quantity rather than altering pore size distribution patterns. Further lowering activator modulus accelerates early densification of pore structures: the 7 d pore characteristic peak of GFJ-15-4 shifts further left with lower peak intensity relative to GFJ-15-2. Nevertheless, GFJ-15-4 presents an obvious rise of 0.6–0.9 μm macropore peaks at 28 d, which is attributed to microcracks and local expansion induced by secondary pozzolanic reactions under high-alkali environments [25]. Even so, substantial reduction of 0.06–0.6 μm medium pores dominates the total porosity evolution, leading to continuous porosity decline and improved macroscopic mechanical properties with curing age.

### 4.2. XRD Mineral Phase Analysis

**Figure 11:**
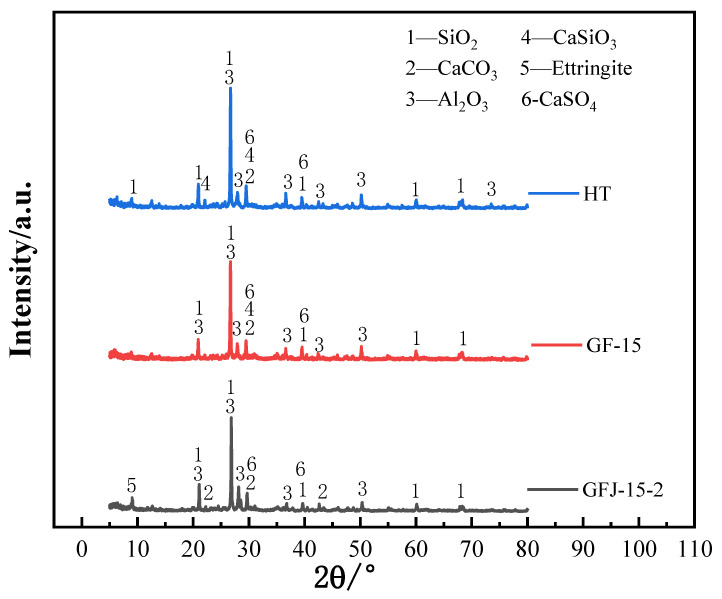
XRD mineral diffraction patterns of raw loess and stabilized loess.

XRD patterns of raw loess and stabilized loess are shown in Figure 11. Primary mineral phases (SiO_2_, Al_2_O_3_) originating from loess and solid waste persist in all stabilized systems. However, GF-15 displays slightly weakened diffraction peaks at 2θ = 21°, 26°, and 36.6° compared with HT, which indicates partial dissolution of silico-aluminate components via pozzolanic reactions to form amorphous gel phases. The alkali-activated GFJ-15-2 group exhibits further weakened diffraction intensities at 2θ = 26°, 36.6°, and 50°, as more silico-aluminate precursors dissolve and transform into amorphous geopolymers under alkaline activation. A distinct ettringite diffraction peak appears at 2θ = 9° for GFJ-15-2. This originates from ion reactions between free Ca^2+^/Al^3+^ and [SO_4_]^2−^ released from solid waste dissolution, followed by nucleation and crystal growth within alkaline matrices. A new CaCO_3_ diffraction peak is also detected in GFJ-15-2: free Ca^2+^ combines with OH^−^ to precipitate Ca(OH)_2_, which subsequently carbonates with atmospheric CO_2_ to form calcium carbonate crystals.

### 4.3. SEM-EDS Micromorphology Analysis

**Figure 12:**
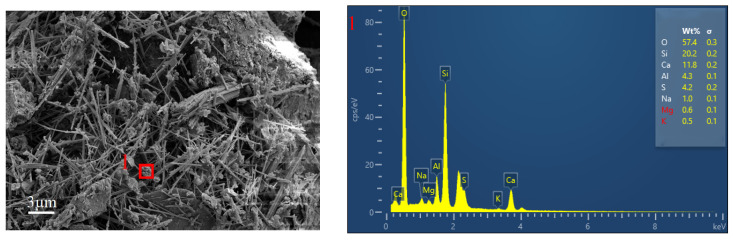

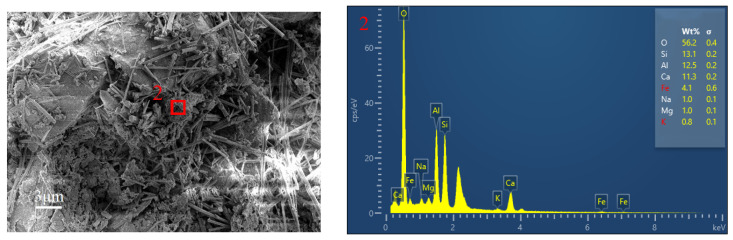
EDS elemental spectra of alkali-activated multi-source solid waste stabilized loess.

Figure 12 presents EDS elemental spectra of alkali-activated multi-source solid waste stabilized loess. Existing geopolymer research establishes that hydration products are dominated by calcium aluminosilicate hydrate (C-A-S-H) gel when the Ca/Si molar ratio exceeds 0.6; mixed C-A-S-H and sodium aluminosilicate hydrate (N-A-S-H) gels form at Ca/Si < 0.6; pure N-A-S-H gel generates as Ca/Si approaches zero [26]. EDS elemental quantification confirms that Region 1 contains mixed C-A-S-H/N-A-S-H gels, while Region 2 mainly consists of C-A-S-H gel. Therefore, the cementitious matrix of alkali-activated TP-FA-GGBS stabilized loess is composed of dual C-A-S-H and N-A-S-H geopolymer gels.

**Figure 13:**
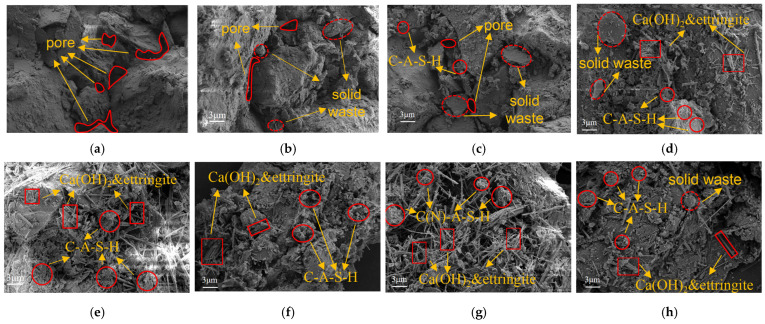
SEM image of stabilized loess: (**a**) HT (**b**) SEM diagram of GF-15 group cured for 7 days (**c**) SEM diagram of GF-15 group cured for 28 days (**d**) SEM diagram of GFJ-15-2 group cured for 7 days (**e**) SEM diagram of GFJ-15-2 group cured for 28 days (**f**) SEM diagram of GFJ-15-4 group cured for 7 days (**g**) SEM diagram of GFJ-15-4 group cured for 28 days (**h**) SEM diagram of GFJ-15-1 group cured for 28 days.

SEM images of raw loess and stabilized loess at different curing ages are displayed in Figure 13. Raw loess (Figure 13a) possesses abundant large intergranular voids and weak interparticle cementation, which fundamentally accounts for its poor geotechnical performance. Figure 13b,c show GF-15 specimens cured for 7 d and 28 d, respectively. At 7 d, solid waste particles fill partial loess voids, yet interparticle bonding remains weak due to insufficient hydration. After 28 d curing, limited hydration products form weak cementation bridges between particles and improve soil densification, though abundant unreacted solid waste particles remain inside voids, indicating incomplete utilization of pozzolanic potential. Comparing Figure 13d–h, alkali activation generates massive hydration gels within the soil matrix. Unreacted solid waste particles gradually decrease while gel products accumulate with extended curing age, further filling intergranular voids and greatly enhancing interparticle bonding strength. The hydration degree and gel yield of GFJ-15-4 at 7 d are comparable to GFJ-15-2 after 28 d curing. GFJ-15-1 (high activator modulus) cured for 28 d contains far fewer hydration products and more residual solid waste particles relative to GFJ-15-2, proving that reducing activator modulus accelerates solid waste pozzolanic reaction kinetics.

### 4.4. Mechanism of Stabilized Loess Action

**Figure 14:**
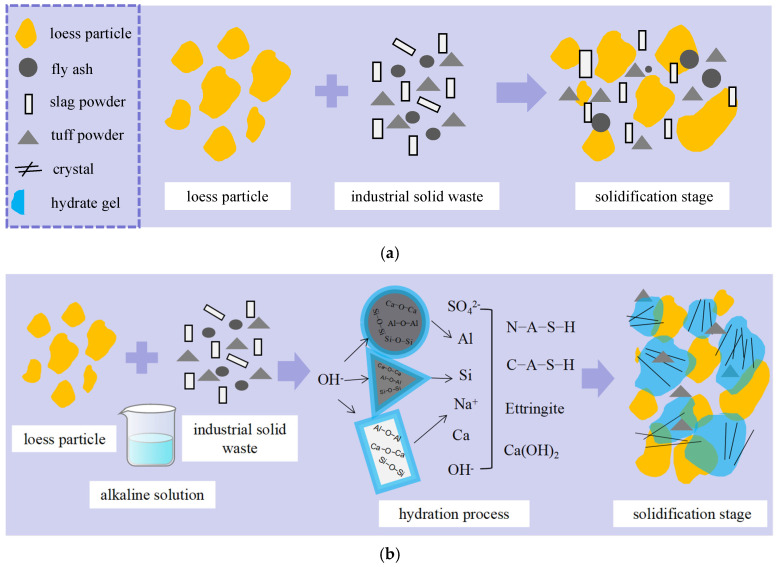
Schematic stabilization mechanism: (**a**) Stabilization mechanism of non-activated multi-source solid waste-stabilized loess (**b**) Reaction mechanism of alkali-activated multi-source solid waste stabilized loess.

Combining macroscopic mechanical tests and multi-scale microcharacterization, the stabilization mechanism of multi-source solid waste stabilized loess can be summarized into three coupled effects: (1) Physical void filling of fine solid waste particles; (2) Alkaline activation accelerating pozzolanic reactions of solid waste active phases; (3) Cementation reinforcement induced by hydration gels between soil particles. The full reaction schematic is provided in Figure 14. As illustrated in Figure 14a, multi-source solid waste particles fill loess intergranular voids and improve soil compactness via physical filling. Nevertheless, the low inherent reactivity of unactivated solid waste leads to slow hydration kinetics, limited cementitious product generation, weak interparticle bonding, delayed early strength gain, and poor water resistance. Figure 14b demonstrates the reaction pathway under dual NaOH-Na_2_SiO_3_ activation. NaOH provides a high-alkaline environment, where abundant OH^−^ breaks Si-O and Al-O bonds on solid waste particle surfaces, depolymerizing aluminosilicate glass networks and releasing reactive silicate/aluminate monomers. These monomers react with Ca^2+^ and Na^+^ in pore solution to precipitate C-A-S-H and N-A-S-H geopolymer gels [27]. Sodium silicate dissociates into Na^+^ and [SiO_3_]^2−^ while releasing extra OH^−^ to sustain matrix alkalinity, further promoting glass phase dissolution. Dissolved [SiO_3_]^2−^ directly participates in gel formation with Ca^2+^/Na^+^, and free Ca^2+^ precipitates as Ca(OH)_2_ crystals via combination with OH^−^ [28]. Free Al^3+^, Ca^2+^ and [SO_4_]^2−^ undergo ionic crystallization to form needle-shaped ettringite, jointly constructing a continuous cementitious spatial network. Compared with conventional FA-GGBS alkali-activated systems, the TP-FA-GGBS ternary blend exhibits unique advantages stemming from TP’s dual filling and nucleation effects. Fine TP particles reduce overall porosity of stabilized loess to elevate mechanical performance; simultaneously, TP acts as heterogeneous nucleation sites for hydration product crystallization to accelerate gel formation and optimize pore structure evolution [29]. Synergistic physical filling and chemical cementation jointly improve the compactness and long-term stability of stabilized loess.

## 5. Carbon Emission Assessment

The global warming potential over a 100-year time horizon (GWP_100_) was employed to quantify greenhouse gas emissions from stabilized loess, whereby all greenhouse gas species were converted into equivalent carbon dioxide mass. To unify the accounting boundary, a unified transportation distance of 50 km was adopted for all raw materials. Heavy-duty diesel trucks with a rated load capacity of 30 tons were selected as the transportation equipment. The 6% cement dosage was selected as the reference benchmark for comparative analysis, which corresponds to the typical cement mixing proportion widely adopted in geotechnical engineering and practical subgrade improvement projects; this detailed rationale has been supplemented in the revised manuscript to accommodate readers of materials science journals. Carbon emission reduction effects of loess stabilized with multi-source solid wastes were evaluated by comparison with 6% cement-stabilized loess, and the carbon emission factors of all raw materials are tabulated in Table 5. TP is a by-product generated during manufactured sand processing, with nearly zero carbon emissions arising from its production stage [30]. Raw loess involves no manufacturing-related emissions, so only transportation-induced carbon costs were accounted for both raw loess and TP. Total carbon emissions per ton of stabilized loess specimens are summarized in Table 6.

The total carbon footprint of loess stabilized with 6% cement reaches 53.72 kg CO_2_ eq per ton. For mixtures stabilized with unactivated multi-source solid wastes, total carbon emissions merely range from 5.96 to 9.92 kg CO_2_ eq/t, delivering carbon reduction rates of 81.5–88.9% relative to cement-stabilized loess. Alkali-activated composite samples exhibit carbon emissions of 7.28–13.92 kg CO_2_ eq/t. Although alkaline activators bring additional carbon loads, their overall carbon footprints are still 74.1–86.4% lower than that of cement-stabilized loess.

Decomposition of carbon emission components indicates that ground granulated blast-furnace slag (GGBS) and alkaline activators dominate the total emissions of alkali-activated stabilized loess. Despite its moderate emission factor of 62.4 kg CO_2_ eq/t, the high mixing dosage of GGBS accounts for a substantial proportion of total carbon outputs. Sodium hydroxide (NaOH) and sodium silicate exhibit extremely high emission factors (461 kg CO_2_ eq/t and 735 kg CO_2_ eq/t, respectively), second only to cement. Nevertheless, their low mixing fractions guarantee outstanding overall carbon reduction performance. The dosage of alkaline activators shows a strong correlation with mechanical properties. Among all tested formulations, the GFJ-15-3 group achieves optimal geotechnical performance with total carbon emissions of 13.39 kg CO_2_ eq/t, corresponding to a 75.1% carbon reduction compared with the 6% cement reference group.

## 6. Conclusions

- Incorporating multi-source solid waste slightly raises the optimum moisture content while significantly increasing the maximum dry density of stabilized loess; both compaction indices peak at a solid waste dosage of 15%. Non-activated solid waste improves soil strength, and water resistance but suffers poor early performance, which can be effectively remedied by alkali activation. Reducing activator modulus accelerates early strength gain and pore refinement, yet excessively low modulus triggers secondary expansion and macropore development in later curing stages. The optimal mix formulation consists of 15% ternary solid waste blend, 3% NaOH and 3.2% Na_2_SiO_3_ (relative to solid waste mass).
- Both non-activated and alkali-activated multi-source solid waste blends improve the compressive strength, shear strength, and water stability of loess. Non-activated solid waste stabilized loess exhibits slow early strength development and extremely weak water resistance. Alkali activation remarkably accelerates early strength evolution and greatly elevates early compressive strength, shear strength and water resistance. Lower activator modulus further promotes early mechanical performance. The formulation with 15% solid waste, 3% NaOH and 3.2% Na_2_SiO_3_ achieves the optimal comprehensive mechanical properties.
- Multi-source solid waste (activated or non-activated) reduces soil porosity, and total porosity gradually declines with curing age. Decreasing activator modulus optimizes early pore structures, whereas excessively low modulus may induce pore expansion in late curing periods.
- The stabilization mechanism relies on two synergistic effects: physical void filling of fine solid waste particles and chemical alkali-activated cementation. Alkaline activators accelerate solid waste pozzolanic reactions to generate C-A-S-H/N-A-S-H geopolymer gels, ettringite, and Ca(OH)_2_ crystals, forming continuous cementation networks to bind soil particles. Combined physical filling and chemical gelation collectively enhance the overall geotechnical performance of loess.

Multi-source solid waste stabilized loess delivers prominent carbon reduction benefits. Compared with conventional cement-stabilized loess, carbon emissions decrease by 74.1–88.9%. The optimal mechanical formulation (GFJ-15-3) cuts carbon emissions by 75.1%.

The long-term durability test of loess stabilized with multi-source solid wastes was not conducted in this study, which is a limitation of the present research. The research team will carry out in-depth research on this part of content in subsequent studies. In addition, during the carbon emission calculation process, the carbon emission values generated during the production of sodium hydroxide and sodium silicate were not taken into consideration, which may lead to the overestimation of the environmental benefits of the developed materials.

## Figures and Tables

**Table 1:** Physical indices of raw loess.

Particle Density/(g·cm^−3^)	Liquid Limit/%	Plastic Limit/%	Plasticity Index	Maximum Dry Density/(g·cm^−3^)	Optimum Moisture Content/%
2.68	25.2	16.4	8.8	1.75	12.5

**Table 2:** Chemical oxide compositions of solid waste and raw loess (mass fraction, %).

Material	SiO_2_	Al_2_O_3_	Fe_2_O_3_	CaO	MgO	Na_2_O	K_2_O	SO_3_	LOI
TP	47.40	18.60	12.30	12.20	4.88	1.47	1.19	0.02	5.71
FA	51.45	29.51	5.54	5.34	1.37	1.59	2.01	0.69	4.3
GGBS	35.47	15.34	0.45	35.38	8.34	0.67	0.34	2.31	0.7
Loess	61.05	14.62	5.80	11.64	2.29	—	3.19	0.41	6.7

**Table 3:** Mix Proportion.

Group ID	Loess/kg	Water/kg	TP/kg	FA/kg	GGBS/kg	NaOH/kg	Na_2_SiO_3_/kg	Modulus
HT	100	12.50	—	—	—	—	—	—
GF-5		13.13	1.0	1.0	3.0	—	—	—
GF-8		13.50	1.6	1.6	4.8	—	—	—
GF-10		14.30	2.0	2.0	6.0	—	—	—
GF-12		14.56	2.4	2.4	7.2	—	—	—
GF-15		14.95	3.0	3.0	9.0	—	—	—
GF-20		15.00	4.0	4.0	12.0	—	—	—
GFJ-5-2		13.13	1.0	1.0	3.0	0.10	0.16	0.512
GFJ-8-2		13.50	1.6	1.6	4.8	0.16	0.26	0.512
GFJ-10-2		14.30	2.0	2.0	6.0	0.20	0.32	0.512
GFJ-12-2		14.56	2.4	2.4	7.2	0.24	0.38	0.512
GFJ-15-1		15.60	3.0	3.0	9.0	0.15	0.48	0.677
GFJ-15-2		15.60	3.0	3.0	9.0	0.30	0.48	0.512
GFJ-15-3		15.60	3.0	3.0	9.0	0.45	0.48	0.412
GFJ-15-4		15.60	3.0	3.0	9.0	0.60	0.48	0.340

Note: 1. The quality of the water in the table is the water requirement for the compaction test to obtain the optimal water content. 2. HT represents loess in the table; GF represents solid waste; GFJ represents alkali-activated solid waste; the number in the table is ‘improved method-solid waste dosage-NaOH dosage’.

**Table 4:** Compaction results of stabilized loess with different solid waste dosages.

Group ID	Optimum Moisture Content/%	Maximum Dry Density/(g·cm^−3^)
HT	12.5	1.75
GF-5	12.59	1.85
GF-8	12.65	1.85
GF-10	12.63	1.88
GF-12	12.68	1.89
GF-15	12.69	1.89
GF-20	11.98	1.87

**Table 5:** Carbon emission factors of raw materials (GWP, kg CO_2_ eq/t).

Material	Cement [31]	TP [32]	FA [33]	GGBS [32]	NaOH [34]	Na_2_SiO_3_ [35]	Waste [34]	Loess [32]
GWP	930	4.86	8.0	62.4	461	735	0.91	4.86

**Table 6:** Total carbon emissions per ton of stabilized loess (kg CO_2_ eq/t).

Group ID	Cement	TP	FA	GGBS	NaOH	Na_2_SiO_3_	Waste	Loess	Total Emissions
SN-6	49.3	-	-	-	-	-	0.1	4.32	53.72
GF-5	-	0.04	0.07	1.63	-	-	0.10	4.12	5.96
GF-8	-	0.06	0.11	2.56	-	-	0.10	4.03	6.86
GF-10	-	0.08	0.13	3.10	-	-	0.10	3.91	7.32
GF-12	-	0.09	0.15	3.70	-	-	0.11	3.89	7.94
GF-15	-	0.11	0.18	4.45	-	-	0.11	3.74	8.59
GF-20	-	0.15	0.24	5.78	-	-	0.10	3.65	9.92
GFJ-5-2	-	0.04	0.07	1.58	0.39	0.99	0.10	4.11	7.28
GFJ-8-2	-	0.06	0.10	2.46	0.60	1.54	0.10	3.99	8.85
GFJ-10-2	-	0.08	0.13	3.00	0.74	1.89	0.10	3.90	9.84
GFJ-12-2	-	0.09	0.15	3.54	0.87	2.22	0.10	3.82	10.79
GFJ-15-1	-	0.11	0.18	4.30	0.53	3.41	0.10	3.73	12.36
GFJ-15-2	-	0.11	0.18	4.30	1.06	3.41	0.10	3.72	12.88
GFJ-15-3	-	0.11	0.18	4.30	1.58	3.41	0.10	3.71	13.39
GFJ-15-4	-	0.11	0.18	4.30	2.11	3.41	0.10	3.71	13.92

## Data Availability

The original contributions presented in this study are included in the article. Further inquiries can be directed to the corresponding author.

## Abbreviations

The following abbreviations are used in this manuscript:

FA	Fly ash
TP	Tuff powder
GGBS	Ground granulated blast-furnace slag
XRD	X-ray diffraction
SEM	Scanning electron microscopy
NMR	Nuclear magnetic resonance
GWP	Global warming potential
C-A-S-H	Calcium aluminosilicate hydrate
N-A-S-H	Sodium aluminosilicate hydrate
